# Body size preferences and food choice among mothers and children in Malawi

**DOI:** 10.1111/mcn.13024

**Published:** 2020-07-07

**Authors:** Valerie L. Flax, Chrissie Thakwalakwa, John C. Phuka, Lindsay M. Jaacks

**Affiliations:** ^1^ Public Health Research Division RTI International Research Triangle Park North Carolina USA; ^2^ Centre for Social Research, Chancellor College University of Malawi Zomba Malawi; ^3^ College of Medicine University of Malawi Blantyre Malawi; ^4^ Department of Global Health and Population Harvard T.H. Chan School of Public Health Boston Massachusetts USA

**Keywords:** body size perceptions, child, food choice, Malawi, mother, overweight, sub‐Saharan Africa

## Abstract

Overweight in mothers and children in sub‐Saharan Africa is rapidly increasing and may be related to body size perceptions and preferences. We enrolled 268 mother–child (6–59 months) pairs in central Malawi; 71% of mothers and 56% of children were overweight/obese, and the remainder were normal weight. Interviewers used seven body silhouette drawings and a questionnaire with open‐ and closed‐ended questions to measure mothers' perceptions of current, preferred and healthy maternal and child body sizes and their relation to food choices. Overweight/obese and normal weight mothers' correct identification of their current weight status (72% vs. 64%), preference for overweight/obese body size (68% both) and selection of an overweight/obese silhouette as healthy (94% vs. 96%) did not differ by weight status. Fewer overweight/obese than normal weight mothers' preferred body silhouette was larger than their current silhouette (74% vs. 29%, *p* < .001). More mothers of overweight than normal weight children correctly identified the child's current weight status (55% vs. 42%, *p* < .05) and preferred an overweight/obese body size for the child (70% vs. 58%, *p* < .01), and both groups selected overweight/obese silhouettes as healthy for children. More than half of mothers in both groups wanted their child to be larger than the current size. Mothers said that increasing consumption of fruits, vegetables, meat, milk, grains, fizzy drinks and fatty foods could facilitate weight gain, but many cannot afford to purchase some of these foods. Their desired strategies for increasing weight indicate that body size preferences may drive food choice but could be limited by affordability.

**Key messages**

- Mothers more accurately identified their own body size than their children's. Estimates for their children were more accurate if the child was overweight/obese.
- Mothers preferred overweight/obese body sizes for themselves and for their children, especially if the child was already overweight/obese. Nearly all mothers said a large body size was healthy.
- Mothers described a mix of healthy (e.g., fruits, vegetables and milk) and unhealthy (e.g., fizzy drinks and sweetened yogurt) foods that they could eat or feed their child to facilitate weight gain.
- Double duty messages emphasizing healthy foods and foods that contribute to overweight/obesity among mothers and children are needed. Culturally competent communication about healthy and unhealthy body sizes is also needed.

## 1 INTRODUCTION

Globally, two billion adults and 38 million children under 5 years of age are overweight or have obesity (Development Initiatives, 2018). Overweight and obesity are risk factors for non‐communicable diseases, which account for approximately three quarters of mortality worldwide (GBD 2017 Causes of Death Collaborators, 2018). In sub‐Saharan Africa, overweight and obesity are on the rise, especially in urban areas and among women, but also increasingly in rural areas and among young children (BeLue , 2009; Jaacks et al., 2019; Jaacks, Slining, & Popkin, 2015; Tzioumis, Kay, Bentley, & Adair, 2016). Urbanization, sedentary lifestyles, excess food intake and changes in food systems have contributed to increases in overweight and obesity in sub‐Saharan Africa but do not fully explain these increases (NCD Risk Factor Collaboration, 2019; Popkin, Adair, & Ng, 2012; Wallace et al., 2019). Sociocultural factors, including cultural norms related to body size perceptions and preferences, may also play a role (Benkeser, Biritwum, & Hill, 2012; Holdsworth, Gartner, Landais, Maire, & Delpeuch, 2004; Popenoe, 2004; Walentowitz, 2011). In sub‐Saharan Africa, large body sizes are preferred, especially for women, because they are linked with good health, beauty, fertility and wealth (Appiah, Otoo, & Steiner‐Asiedu, 2016; Devanathan, Esterhuizen, & Govender, 2013; Draper, Davidowitz, & Goedecke, 2016; Holdsworth et al., 2004; Matoti‐Mvalo & Puoane, 2011; Muhihi et al., 2012; Tateyama et al., 2019). This preference is further fuelled where HIV is prevalent because being thin is considered a sign of HIV (Croffut et al., 2018; Muhihi et al., 2012; Tateyama et al., 2019).

Mothers' perceptions of their own and their child's body size could influence or be driven by their body size preferences. Studies of adults in sub‐Saharan Africa have shown that many individuals misperceive their current body size and tend to underestimate it, particularly if they are overweight or obese (Akinpelu, Oyewole, & Adekanla, 2015; Devanathan et al., 2013; Muhihi et al., 2012; Tateyama et al., 2018). A meta‐analysis of 78 samples in high‐ and middle‐income countries found that about 50% of parents underestimated their child's weight status (Lundahl, Kidwell, & Nelson, 2014). In these settings, mothers are more likely to underestimate their child's weight when the child is overweight or has obesity (Francescatto, Santos, Coutinho, & Costa, 2014), and parents who prefer a larger body size for their child tend to underestimate their child's weight status more than parents who prefer a lean child body size (Pasch et al., 2016). We identified only one study of child body size preferences in sub‐Saharan Africa, conducted in Nigeria. It showed that 35% of mothers underestimated and 24% overestimated their child's body size (Adeniyi, Ekure, Olatona, Ajayi, & Nworgu, 2018).

The ability of mothers to accurately estimate their own body size and that of their child and their preferences for certain body sizes may influence food choices for individuals and families. For example, if a mother perceives herself or her child to be thin, even if they are overweight, but her preference is for a larger body size, her perceptions may play a role in the types and quantities of food she buys for herself and her family and the frequency and quantity of their food consumption. However, there is little research to date on the link between body size perceptions or preferences and food choice. In addition, there is limited research on maternal perceptions of child body size in sub‐Saharan Africa (Adeniyi et al., 2018) and the association of maternal body size with mothers' preferences for child body size. This formative research study was conducted with the aim of understanding Malawian mothers' body size perceptions and preferences for themselves and their child and the relationship of these perceptions and preferences with food choices. Our overall goal was to provide information for the design of public health programmes and policies in Malawi to address the growing problem of overweight among mothers and children.

## 2 METHODS

### 2.1 Setting

This analysis uses data from a study of drivers of food choice in households where the mother, child or both were overweight. The study was conducted in Lilongwe and Kasungu Districts in central Malawi. We selected these areas because they have a higher prevalence of overweight/obesity among mothers and children younger than 5 years of age than other parts of Malawi (National Statistical Office [NSO] Malawi & ICF, 2017). In each district, we chose two urban neighbourhoods and two rural communities as data collection sites.

### 2.2 Study population

At each site, mothers with children 6 months to 5 years of age were invited for screening at a central location. Five research assistants were trained to collect anthropometric measurements (Cogill, 2003), and their measurements were standardized against those of an experienced researcher prior to starting data collection. Maternal height and standing height of children 2 years or older were measured to the nearest 0.1 cm using a portable stadiometer (Seca 213). Recumbent length of children younger than 2 years was measured to the nearest 0.1 cm using an infant measuring mat (Seca 210). Weight of mothers and children 2 years or older was measured to the nearest 0.1 kg using a digital scale (Seca 803). Weight of children younger than 2 years was measured to the nearest 0.1 kg using a digital baby scale (Seca 354). We used the anthropometric data to calculate body mass index (BMI, kg/m^2^) of mothers and used the standard cut‐offs for normal weight (18.5 kg/m^2^ ≤ BMI < 25 kg/m^2^) and overweight or obesity (BMI ≥ 25 kg/m^2^). We calculated weight‐for‐height*z*‐scores(WHZ) for children using the World Health Organization (WHO) growth standard and used the WHO cut‐offs for normal weight (−2 SD < WHZ ≤ +2 SD) and overweight (WHZ > +2 SD; WHO Multicentre Growth Reference Study Group, 2006). Mother–child dyads were purposefully enrolled in three groups: overweight mother with an overweight child, overweight mother with a normal weight child and normal weight mother with an overweight child. This purposive sampling technique was used to ensure that we had sufficient representation to draw inferences about the relationship between maternal/child weight status, and body size perceptions and preferences, as well as food choice.

### 2.3 Data collection procedures

**FIGURE 1:**
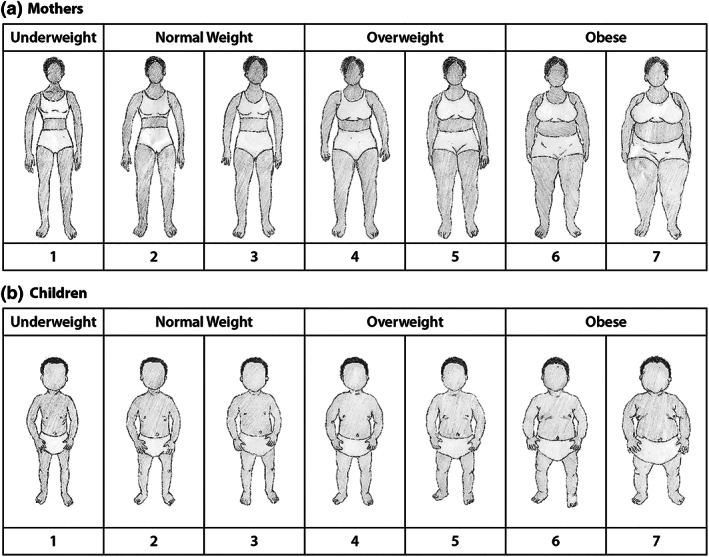
Mother and child body silhouettes

Research assistants were trained to use a set of seven adult female and seven child body silhouette drawings (Figure 1) and a semistructured questionnaire to measure mothers' perceptions of their current, preferred and healthy body sizes for themselves and their child. A local artist adapted mothers' body silhouettes from a version previously used in Malawi (Bentley et al., 2005; Croffut et al., 2018) and validated in a sample of mother–daughter dyads in South Africa (McIza et al., 2005 ). The same artist adapted child body silhouettes from Hager, McGill, and Black (2010). Both the mother and child silhouettes were originally patterned on Stunkard, Sorensen, and Schulsinger (1983). For the mothers' silhouettes, we followed a similar BMI categorization as a previous study that developed body silhouettes for an African American population (Pulvers et al., 2004), later validated in a sample of women in the Seychelles (Yepes, Viswanathan, Bovet, & Maurer, 2015). In this study, the thinnest silhouette was assigned a BMI of 17 kg/m^2^ with a 3‐BMI unit increment for each subsequent silhouette, making the heaviest silhouette equal to 35 kg/m^2^. Consequently, Silhouette 1 was classified as underweight, Silhouettes 2 and 3 as normal weight, Silhouettes 4 and 5 as overweight and Silhouettes 6 and 7 as obese. We applied the same categorization to the children's silhouettes.

Each body silhouette drawing was printed separately on cardstock and laminated. The interviewer mixed the body silhouettes and laid them out in a random order before each question (i.e., current, preferred and healthy body size) separately for the mother to make selections for herself and then again for her child. The specific questions related to selection of the silhouettes for women were as follows:

1. Please look at these drawings of women and tell me which figure most closely resembles your figure. (Current body size)
2. Which figure would you want your figure to look like? (Preferred body size)
3. Which figure do you think shows a healthy woman? (Healthy body size)

Open‐ended questions in the questionnaire were used to understand mothers' body size selections and how they were related to food choices. Questions about food choice included:

- If the answers to Questions 1 and 2 were different, ask:
  How does the difference between your current figure and the figure you would like to have influence what types of food and drinks you buy?
- If the answers to Questions 1 and 3 were different, ask:
  How does the difference between your current figure and the figure you believe is healthy influence what types of food and drinks you buy?
- What types of food or drinks will help you get to (or stay at) the figure you would like to have? Probe: How will these types of food and drinks help you do that? How often are you able to buy them? What keeps you from buying them more often?

A similar set of questions were used to obtain information about mothers' preferences for child body size and food choices.

### 2.4 Data analysis

We performed chi‐squared tests comparing body size perceptions to actual body size by normal versus overweight status separately for mothers and children. We calculated the difference between the selected current and preferred body silhouette numbers for mothers and children to quantify how many preferred a smaller, the same or a larger body size and conducted chi‐squared tests comparing the difference by mother and child weight status. We also used chi‐squared tests to examine differences in the mother's selections of child current, preferred and healthy body silhouettes by the mother's weight status. We calculated descriptive statistics for participants' socio‐economic characteristics, including age, maternal education (secondary or higher vs. primary or no education), household assets (sum of 12 household durable goods, range 0–12), household food insecurity access score (HFIAS) as a continuous variable (range 0–27; Coates, Swindale, & Bilinksy, 2007) and rural/urban residence.

We sorted responses to open‐ended questions in an Excel data matrix by weight status based on BMI or WHZ. After reading through the responses, we developed codes based on the main topics that emerged from each open‐ended question. Within the sorted segments of the open‐ended data, one researcher applied the codes and summarized the findings using qualitative content analysis methods (Hsieh & Shannon, 2005). For lists of foods provided in open‐ended responses, we divided the data for mothers and children by those whose preferred silhouette was smaller than their current silhouette and those whose preferred silhouette was the same or larger than their current silhouette. We then tabulated the food items and ranked the top 10 foods from most to least frequently mentioned.

### 2.5 Ethical considerations

Participants received an incentive of 4 US dollars. The study was approved by the College of Medicine Research Ethics Committee at the University of Malawi and by the institutional review boards at RTI International and the Harvard T.H. Chan School of Public Health.

## 3 RESULTS

We included 268 mother–child pairs in this analysis. Seventy‐eight mothers (29%) were normal weight, and 190 (71%) were overweight/obese; 118 children (44%) were normal weight, and 150 (56%) were overweight/obese. Their socio‐economic characteristics are shown in Table 1.

**TABLE 1:** Participant characteristics (*N* = 268 mother–child pairs)

	*N (%)*
Mother's weight status	
Normal weight	78 (29)
Overweight or obese	190 (71)
Overweight	126 (47)
Obese	64 (24)
Child's weight status	
Normal weight	118 (44)
Overweight or obese	150 (56)
Overweight	132 (49)
Obese	18 (7)
Mother's level of education	
Primary or lower	156 (58)
Secondary or higher	112 (42)
Location of residence	
Urban	134 (50)
Rural	134 (50)
	Mean ± SD
Mother's age, years	28.3 ± 6.6
Child's age, months	27.0 ± 15.4
Asset index (12 items)	3.9 ± 3.0
Household food insecurity access score (range 0–27)	5.5 ± 7.0

### 3.1 Maternal body size perceptions and preferences

**FIGURE 2:**
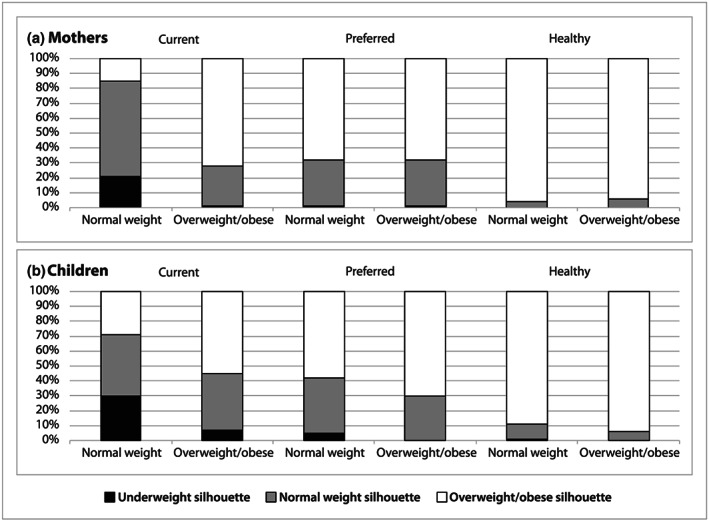
Current, preferred and healthy mother and child body silhouette selections by weight status (Mothers: normal weight, *N* = 78, overweight/obese, *N* = 190; Children: normal weight, *N* = 118, overweight/obese, *N* = 150)

We found no significant differences by weight status in mothers' selections of body silhouettes representing their current, preferred and healthy body size (Figure 2a). Overall, 70% of mothers chose a body silhouette that correctly represented their weight status (72% overweight/obese vs. 64% normal weight mothers, *p* = .195), and 26% underestimated their weight status (28% overweight/obese and 21% normal weight mothers). About two thirds of mothers selected an overweight/obese silhouette as their preferred size (68% in both groups, *p* = .985), and nearly all mothers selected an overweight/obese silhouette as a healthy size (94% overweight/obese vs. 96% normal weight, *p* = .424), with no significant differences in either of these outcomes by weight status. Half of all mothers selected the largest body silhouette as healthy.

**FIGURE 3:**
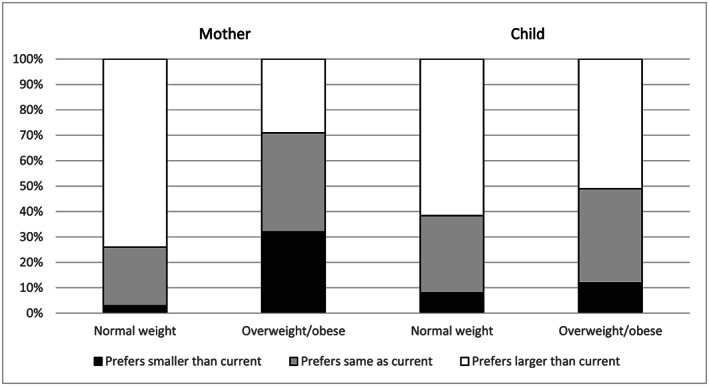
Difference in mothers' selections of preferred versus current body silhouettes for herself and her child by maternal and child weight status (Mothers: normal weight, *N* = 78, overweight/obese, *N* = 190; Children: normal weight, *N* = 118, overweight/obese, *N* = 150)

Significantly more normal weight than overweight/obese mothers wanted a larger body size based on the difference between their silhouette selections for preferred and current sizes (73% normal weight vs. 29% overweight/obese mothers, *p* < .001; Figure 3). Approximately one quarter of the mothers preferred a silhouette that was smaller than their current silhouette. Nearly all of these mothers were overweight (45%) or obese (52%) and tended to be based in urban areas (71%) and have a secondary education or higher (70%).

Mothers who preferred overweight/obese body sizes explained that they chose the larger silhouettes because they show a woman who is healthy and eats well. For example, a normal weight mother (ID 92) who preferred Silhouette 5 said, ‘I want to look fat and people should know that I eat well’. An overweight mother (ID 124) who preferred Silhouette 6 explained, ‘The woman [with a large body] looks strong and healthy. It seems she cannot fall sick and she can work the way she wants’.

Among the mothers who preferred a normal weight body silhouette or whose preferred body silhouette was smaller than their selected current silhouette, most said they wanted a normal weight body because they associate large body sizes with physical and health issues. An overweight mother (ID 155) who preferred Silhouette 2 said, ‘I want to get slimmer. [When you are heavy] you get tired when walking and you look older than your age’. A normal weight mother (ID 321) who preferred Silhouette 3 explained, ‘Having a big body makes you get diseases like [high] blood pressure and sugar, so a medium one is fine’. Medium was defined most often as Silhouettes 3 through 5, which represents the middle of our silhouette range but includes one normal weight silhouette (3) and two overweight silhouettes (4 and 5).

Mothers who wanted to maintain their body size or become larger (*N* = 205) listed a variety of foods they could eat to achieve this. Some talked about specific foods that could help them do this, whereas others talked about eating a variety of foods to gain or maintain weight and be healthy. The most common foods mothers mentioned were green leafy vegetables (64%), fruits (53%), meat (44%), *nsima* (staple food, stiff maize porridge; 37%), milk (33%), fizzy drinks (soda; 31%), porridge (27%), beans (24%), *maheu* (sweetened maize drink; 20%) and groundnuts or foods containing groundnuts (19%; Table 2). Mothers considered meat to be a fatty food. An overweight mother explained, ‘Fatty foods and fizzy drinks are best for making you fat’. Similarly, a normal weight mother (ID 322) who wanted to become larger said, ‘I should be eating meat, sausage, *maheu*, and fruits’ to gain weight. An overweight mother (ID 323) who wanted to increase her body size said,

**TABLE 2:** Top 10 ranked foods mothers said would help them or their child attain or maintain their preferred body size

Rank	Mothers who want a smaller body size (*N* = 63)		Mothers who want the same or larger body size (*N* = 205)^a^		Mothers who want their child to have a smaller body size (*N* = 28)		Mothers who want their child to have the same or larger body size (*N* = 240)^b^	
	Food	*N* (%)	Food	*N* (%)	Food	*N* (%)	Food	*N* (%)
1	Green leafy vegetables	20 (32)	Green leafy vegetables	131 (64)	Porridge	15 (54)	Porridge	153 (64)
2	Less fats/oil	19 (30)	Fruit	108 (53)	Fruit, *nsima*, fizzy drinks	10 (36)	Fruit	113 (47)
3	Lemons	15 (24)	Meat	91 (44)	*Maheu*	9 (32)	Milk	94 (39)
4	*Nsima*	13 (21)	*Nsima*	75 (37)	Yogurt	8 (29)	*Nsima*	91 (38)
5	Fruit (not lemons)	12 (19)	Milk	68 (33)	Meat, green leafy vegetables	7 (25)	Green leafy vegetables	84 (35)
6	Lots of water, milk	9 (14)	Fizzy drinks	64 (31)	Groundnuts	5 (18)	Meat	67 (28)
7	Fish, meat	8 (13)	Porridge	56 (27)	Milk, rice	4 (14)	Groundnuts	65 (27)
8	Fizzy drinks, juice, food with bicarbonate of soda	7 (11)	Beans	49 (24)	Less fats/oil, juice	3 (11)	Yogurt	63 (26)
9	Less meat/lean meat	6 (10)	*Maheu*	40 (20)	Beans, potatoes, *thobwa*, margarine	2 (7)	Fizzy drinks, *maheu*	61 (25)
10	Beans, porridge, *thobwa*	5 (8)	Groundnuts/groundnut flour	39 (19)	‐	‐	Rice	35 (15)

*Note*: *Porridge* includes porridge made of maize, soya, maize and soya mixed or rice. Porridge containing soya was mentioned most often as facilitating weight gain, especially among children. *Fruit* includes bananas, pawpaws, mangoes, guavas, oranges, tangerines and apples. Lemons were mentioned only by women who wanted to have a smaller body size and were not counted in the fruit category. *Meat* includes beef, goat, pork and chicken. *Yogurt* includes liquid yogurt and thicker yogurt, both of which are usually sweetened and commonly fed to children. *Groundnuts* include plain or roasted groundnuts, groundnut flour and peanut butter. *Nsima* is stiff maize porridge, the staple food. *Maheu* is a sweetened mass‐produced maize drink. *Thobwa* is a fermented maize drink, often home‐produced.

a Ninety‐two mothers wanted to maintain their body size; 113 mothers wanted to increase their body size.

b Ninety‐one mothers wanted their child to maintain his or her body size; 149 mothers wanted their child to increase his or her body size.

I would change to eating foods with oil, tomato, and onion and not just boil [them] as I do right now. I would be buying soya porridge with milk added [and] green leafy vegetables with oil [and] tomato so that I reach that point. The food would be of different food groups and thus cause the body to get bigger.

A normal weight mother (ID 199) who wanted a larger body size said that she would:

eat from different food groups—eat fruits, green leafy vegetables, porridge, soybeans, and milk. These foods add vitamins [to the body]. It makes a person healthy. I buy some of these foods. I buy green leafy vegetables every day. Fish, I buy every week. Meat, I do not buy very often because it's expensive.

Many mothers who preferred a larger body size said that eating some of the types of foods that would make them gain weight, like meat, was not feasible because of their cost. A normal weight mother (ID 282) explained, ‘I can't afford to change what I eat because I do not have money for better foods’.

Mothers who wanted a smaller body size (*N* = 63), nearly all of whom were overweight/obese, described ways they could lose weight, such as eating foods with less fat or oil (30%), eating lemons or adding lemon juice to food (24%), drinking lots of water (14%), eating food with bicarbonate of soda added (e.g., dishes made with okra; 11%) and eating less meat or lean meat (10%; Table 2). They also listed foods like green leafy vegetables (32%); *nsima* (21%); fruit (other than lemons; 19%); milk (14%); fish and meat (13%); fizzy drinks and juice (11%); and beans, porridge and *thobwa* (fermented maize drink; 8%) as foods that would help them attain a smaller body size (Table 2). These lists of foods sometimes differed from the themes that emerged from open‐ended responses. Milk, meat and fizzy drinks were listed by some mothers as foods they would eat to lose weight, but more mothers who wanted a smaller body size described them as foods to avoid. For example, an overweight woman (ID 158) explained that she should ‘start consuming less fats and oils. Eat more green leafy vegetables and less milk and meat. This would help me slim up’. Another overweight woman (ID 31) explained, ‘I will be eating less carbohydrates and I will avoid fizzy drinks and Cokes. I buy apples and natural yogurt almost every day because these foods will help me lose weight a bit’.

Mothers' comments about their healthy body size silhouette selections were similar to those of women whose preferred body silhouette was larger than their selected current body silhouette. Both normal weight and overweight/obese mothers mentioned that they chose an overweight/obese silhouette as healthy because the woman looked fat and well fed and they said a large body was desirable.

### 3.2 Child body size perceptions and preferences

Overall, 49% of mothers correctly selected a silhouette that represented their child's weight status. A higher percentage of mothers with an overweight/obese child compared with those with a normal weight child selected a body silhouette that aligned with the child's current weight status (55% overweight/obese vs. 42% normal weight child, *p* = .036; Figure 2b). More mothers of overweight/obese than normal weight children selected an overweight/obese body silhouette as the preferred size for their child (70% overweight/obese vs. 58% normal weight child, *p* = .006). The majority of mothers selected an overweight/obese silhouette as representing a healthy size for a child, with no difference by child weight status (94% overweight/obese vs. 89% normal weight child, *p* = .233). Fifty‐nine percent of mothers selected the largest child silhouette as healthy. Although fewer mothers of overweight/obese than normal weight children preferred a larger body size for their child based on the difference between their silhouette selections for preferred and current sizes, there was no significant difference by child weight status (51% overweight/obese vs. 61% normal weight child, *p* = .268; Figure 3).

We found no significant differences in mothers' selections of child current, preferred and healthy body silhouettes by the mother's weight status. Forty‐seven percent of overweight/obese mothers versus 54% of normal weight mothers selected an overweight/obese current body silhouette for their child (*p* = .297). Sixty‐two percent of overweight/obese mothers versus 72% of normal weight mothers preferred an overweight/obese body silhouette for their child (*p* = .123). Ninety‐one percent of overweight/obese mothers versus 94% of normal weight mothers selected an overweight/obese silhouette as healthy for their child (*p* = .693).

Mothers who preferred an overweight silhouette for their child said that a larger size shows that the child is healthy and strong. The mother of a normal weight child (ID 124) who selected Silhouette 6 as preferred for her child said, ‘The picture [Silhouette 6] looks good and the baby is healthy and doesn't fall sick and plays a lot’. The mother of an overweight child (ID 269) who preferred Silhouette 5 for her child explained, ‘The picture [Silhouette 5] shows a big body baby, which is what everyone wants’.

Some mothers preferred a normal weight body size for their child because they believe that a medium body size is good, or they worried about problems related to overweight. Mothers' selection of what they considered a ‘medium’ body silhouette for their child ranged from Silhouette 1 to 6, but the term ‘medium’ was used most to describe Child Silhouette 3, which represents normal weight. A mother of a normal weight child (ID 155) who preferred Silhouette 3 said, ‘I want my son to look medium, as he is’. A few mothers of overweight children realized that being overweight could be difficult for the child. A mother with an overweight child (ID 264) who preferred Silhouette 2 explained, ‘A child faces many challenges when they are very fat, and it is hard to be active’.

Mothers who wanted their child to maintain or gain weight (*N* = 240) described how they could feed them a variety of foods to achieve this. The most commonly mentioned foods were porridge (64%), fruit (47%), milk (39%), *nsima* (38%), green leafy vegetables (35%), meat (28%), groundnuts (27%), sweetened yogurt (26%), fizzy drinks and *maheu* (25%) and rice (15%; Table 2). They also talked about frequent feeding and feeding larger quantities to increase the child's weight. A mother of a normal weight child (ID 139) who preferred Silhouette 4 said, ‘I try to give her different varieties of foods, *nsima*, milk, *maheu*, and fruits’. A mother of an overweight child (ID 307) who preferred Silhouette 5 said, ‘I give him porridge, Kamba puffs [packaged maize snack], Sobo [locally produced fizzy drink], and chips’. When asked about their reasons for wanting to give their child certain foods, mothers usually said that they help the child gain weight, stay healthy and be strong. They also explained that the foods have vitamins that help with growth. For example, the mother of an overweight child (ID 247) who preferred Silhouette 6 said she gives her son ‘*maheu*, yogurt, juice, soya bean porridge, *nsima*, and vegetables so he will grow fat and will be protected from infections’. Mothers' responses about the frequency of purchasing the foods they said will help the child maintain or gain weight varied widely. Because they lack funds, the majority said they buy the desired foods once or twice a month, irregularly, when they have money or not at all. The mother of an overweight child (ID 254) explained, ‘We do buy [those foods], but it's once in a while due to money problems’.

Mothers who wanted their child to have a smaller body size (*N* = 28) listed foods similar to those who wanted their child to maintain or gain weight, such as porridge (54%); fruit, *nsima* and fizzy drinks (36%); sweetened yogurt (29%); meat and green leafy vegetables (25%); groundnuts (18%); and milk and rice (14%; Table 2). Like mothers who wanted to reduce their own body size, some mothers who wanted their child to have a smaller body size, mentioned foods they should stop giving their child. For example, the mother of an overweight child (ID 249) said, ‘By reducing giving him fatty foods and sometimes just cooking porridge, he can [become] slim’. Several mothers included fizzy drinks among foods that could help a child have a smaller body size. A mother with an overweight child (ID 313) who listed *nsima*, porridge, rice and fizzy drinks as foods to achieve the smaller body size she prefers for her child said, ‘[These] will help him lose weight. I do buy [them] whenever I have money’.

Mothers' reasons for selecting overweight/obese silhouettes as healthy for a child were similar to their reasons for their preferred child body size. They said the children in those silhouettes looked fat, healthy, well taken care of and well fed. For example, the mother of an overweight child (ID 329) who selected Silhouette 7 as healthy explained, ‘The body shows a child who is fed well and very fat, hence she is healthier than others’.

## 4 DISCUSSION

With overweight and obesity increasing in Malawi (NSO Malawi & ICF, 2017; NSO Malawi & ICF Macro, 2011), understanding mothers' perceptions of their own and their child's current, preferred and healthy body sizes, as well as how body size preferences relate to food choice, is important for achieving the Malawi Ministry of Health's goal to reduce the prevalence of overweight and obesity by 5% (Government of Malawi, 2018). The percentage of mothers in our study who correctly estimated their own weight status (70% overall) was higher than in studies in Nigeria, Kenya, the Seychelles, South Africa, Tanzania and Zambia, some of which included both men and women or only overweight/obese adults (Akindele, Phillips, Igumbor, & Useh, 2017; Devanathan et al., 2013; Ettarh, Van de Vijver, Oti, & Kyobutungi, 2013; Muhihi et al., 2012; Tateyama et al., 2018; Yepes et al., 2015). In the present study, the percentage of women who correctly estimated their current size did not differ significantly by measured weight status, in contrast to some other studies, which found that overweight participants were more likely than normal weight participants to underestimate their weight status (Muhihi et al., 2012; Tateyama et al., 2018).

Mothers in our study were less accurate at assessing their child's weight status than their own, with only half correctly identifying their child's weight. The overall percentage correct in our study was similar to findings from a study in Nigeria, which used the same type of silhouettes, and both studies found that mothers were more likely to underestimate their child's weight status if the child was overweight (Adeniyi et al., 2018). These findings fit the pattern in high‐ and middle‐income countries showing that parents are generally inaccurate at assessing child weight status (Doolen, Alpert, & Miller, 2009; Lundahl et al., 2014; Moffat, 2000) and tend to underestimate the weight status of overweight children (Francescatto et al., 2014).

Mothers in the present study preferred larger body sizes for themselves and their child and overwhelmingly selected large, including the largest body silhouette, as healthy. More than half of the mothers wanted their child to be larger than the current size, regardless of the child's current weight status. In contrast, mothers were much more likely to want to be larger than their current size if they were normal weight, and only one third of overweight/obese mothers wanted to be smaller than their current size. This finding is concerning from the perspective of obesity prevention because it suggests that the majority of overweight/obese mothers in this sample from Malawi are still unaware of the consequences of overweight/obesity and are not thinking about or planning to lose weight. This is similar to other sub‐Saharan countries, where few overweight/obese adults are aware of health issues related to overweight/obesity (Muhihi et al., 2012; Tateyama et al., 2018), but in stark contrast to the United States, where, according to the Centers for Disease Control and Prevention, about half of adults 20 years and older report having tried to lose weight in the past 12 months (Martin, Herrick, Sarafrazi, & Ogden, 2018). More research is needed in sub‐Saharan countries to develop behaviour change communication strategies aimed at shifting norms related to body size toward a healthy size that is neither too thin nor too large. This type of normative shift would have to be designed with a great deal of care so as not to overcompensate because evidence from high‐income countries indicates that a thin ideal body image increases women's body size dissatisfaction and may be related to disordered eating (Hawkins, Richards, Granley, & Stein, 2004; Thompson & Stice, 2001).

Our results are consistent with a recent global analysis that found over half of the variation in mean BMI between countries among women can be accounted for by cultural determinants (Wallace et al., 2019). The obesity epidemic cannot be addressed without first addressing cultural norms related to body size. For women in Malawi and other sub‐Saharan countries, large women are considered to be beautiful and healthy (Draper et al., 2016; Holdsworth et al., 2004; Matoti‐Mvalo & Puoane, 2011; Muhihi et al., 2012; Tateyama et al., 2019). In addition, women are expected to gain weight when they get married because it shows that the marriage is going well and their husband is taking care of them (Appiah et al., 2016; Mvo, Dick, & Steyn, 1999). For women who have not gained weight, like many of those in our sample who were normal weight, this is clearly something they strive for. However, some women who are overweight/obese, particularly those with more education living in urban areas, have realized the negative health implications (e.g., high blood pressure and diabetes) and want to lose weight, which could be leveraged in obesity prevention or overweight reduction efforts. In contrast, Malawian mothers value adiposity in their children because they link it with health, happiness and strength (Flax, Thakwalakwa, & Ashorn, 2016). Like mothers in many other parts of the world, they want to have a ‘chubby’ child because it shows that a woman is a good mother and is taking care of her child very well (Martinez, Rhee, Blanco, & Boutelle, 2017; Waldrop, Page, & Bentley, 2016).

One of the key innovations of this study was linking body size perceptions and preferences to food choice, which has been touched on only briefly in previous research in sub‐Saharan Africa (Draper et al., 2016). We found that the types of foods listed by mothers who wanted a smaller body size and those who wanted to maintain or gain weight had some similarities, such as green leafy vegetables and *nsima* at the same rank. However, mothers who wanted to lose weight cited specific foods they should eat (such as lemons) or should avoid (such as less fats/oil) to attain their goal, which were not included on the food lists of mothers who wanted to maintain or gain weight. The types of foods listed by mothers who wanted their child to have a smaller body size compared with those who wanted their child to maintain or gain weight contained the same foods with several of them in a similar relative order. The number of mothers who wanted their child to lose weight was small, so the rank order of foods for that group should be interpreted with caution. Packaged and fried snacks, which are commonly purchased for children, were described by mothers in open‐ended responses but included infrequently in lists of foods. Fizzy drinks and meat appeared on food lists for mothers and children regardless of whether a body size increase or decrease was desired. Fizzy drinks and meat are among the foods that many mothers said they could not purchase often because they are unaffordable, suggesting that the food lists may represent, in part, mothers' ideal food choices.

For mothers who wanted to maintain their body size or to have a larger body size for themselves or their child, the types of foods they listed were a mix of healthy foods, like fruits, vegetables, and milk, and unhealthy foods, like fizzy drinks and sweetened yogurt. They also included some foods that are healthy in moderation, like meat and groundnuts. It is likely that foods like fruits and vegetables were included in their lists because the Malawi Ministry of Health and its partners have promoted the six Malawian food groups (fruits; vegetables; legumes and nuts; animal source foods; fats; and grains, roots and tubers) as healthy, so some women may perceive that eating more of all food groups will make them healthy, which is equated for many of them with having a large body size. The mix of food items included on the women's lists points to the need for nutrition education not only to focus on a balanced diet but also to guide healthy food choices by including messages on foods and beverages that are not healthy and for which intake should be limited. The health system in Malawi has been focused on undernutrition but needs to pivot toward ‘double duty’ nutrition messages that address undernutrition and also work toward preventing overweight/obesity and diet‐relatednon‐communicable diseases (World Health Organization, 2017).

This study had some limitations. Our sample was enrolled in specific mother–child weight status categories and was divided evenly between urban and rural areas. This purposeful enrolment improved the efficiency of sampling and allowed us to make comparisons between normal weight and overweight individuals, which was one of our primary research questions. However, our sample does not represent all women and children in Malawi. Compared with the 2015–2016 Malawi Demographic and Health Survey, a greater percentage of mothers in our study had some secondary education or higher (NSO & ICF, 2017), but our sample does include varied levels of household income (measured by assets) and household food security. In addition to the limitation of our sampling strategy, the exact instrument we used to evaluate body silhouettes was not previously validated in Malawi, so there may be slight discrepancies between the weight classification we assigned the silhouettes and the true weight status of women and children that look like the women and children depicted in the silhouettes. However, these silhouettes were adapted from silhouettes that were validated in South Africa (McIza et al., 2005) and the Seychelles (Yepes et al., 2015), and used previously in Malawi (Bentley et al., 2005; Croffut et al., 2018), thus minimizing the possibility of such errors.

In conclusion, this study showed that Malawian women are fairly accurate at assessing their own weight status but less accurate at estimating their children's. They generally prefer to have larger body sizes for their child, whereas a preference for a larger body size for themselves was more common for normal weight women. Only one third of overweight/obese women wanted a smaller body size, suggesting gaps in knowledge of the adverse health consequences of overweight/obesity in this sample. The types of foods women would like to eat or feed to their child to increase weight are a mix of healthy and unhealthy foods. These findings could contribute to plans for developing interventions for obesity prevention in Malawi to achieve the Ministry of Health's goals. For example, the selection of the largest body silhouette as ‘healthy’ and the range of silhouettes selected as ‘medium’ suggest that programmes could try to increase knowledge about which silhouettes represent healthy body sizes and increase awareness of the negative health consequences of overweight and obesity. Double duty messages emphasizing which foods are healthy and unhealthy for mothers and children are also needed. In addition, more research on the links between body size preferences and food choice is needed in other parts of sub‐Saharan Africa and other low‐income countries. Future research on factors related to body size preferences should be driven by behavioural theory, such as the theory of reasoned action or the transtheoretical model (Ajzen, 1992; Prochaska & Velicer, 1997), to better understand the more proximate attitudes, beliefs and social norms that may be related to preferences and their relationship to food choice and dietary intake.

## CONFLICTS OF INTEREST

The authors declare that they have no conflicts of interest.

## CONTRIBUTIONS

VLF, CT and LMJ designed the study. CT and JCP oversaw data collection. VLF conducted the analysis and drafted the manuscript. All authors contributed to the final version of the manuscript.
